# The role of retrotransposons in gene family expansions: insights from the mouse *Abp* gene family

**DOI:** 10.1186/1471-2148-13-107

**Published:** 2013-05-29

**Authors:** Václav Janoušek, Robert C Karn, Christina M Laukaitis

**Affiliations:** 1 Department of Zoology, Faculty of Science, Charles University in Prague, Viničná 7, Prague 128 43, Czech Republic; 2 Department of Medicine, College of Medicine, University of Arizona, Tucson, AZ, USA

**Keywords:** House mouse, Gene duplication, Androgen-binding protein, LINE1, ERVII, NAHR

## Abstract

**Background:**

Retrotransposons have been suggested to provide a substrate for non-allelic homologous recombination (NAHR) and thereby promote gene family expansion. Their precise role, however, is controversial. Here we ask whether retrotransposons contributed to the recent expansions of the *Androgen*-*binding protein* (*Abp*) gene families that occurred independently in the mouse and rat genomes.

**Results:**

Using dot plot analysis, we found that the most recent duplication in the *Abp* region of the mouse genome is flanked by *L1Md*_*T* elements. Analysis of the sequence of these elements revealed breakpoints that are the relicts of the recombination that caused the duplication, confirming that the duplication arose as a result of NAHR using L1 elements as substrates. L1 and ERVII retrotransposons are considerably denser in the *Abp* regions than in one Mb flanking regions, while other repeat types are depleted in the *Abp* regions compared to flanking regions. L1 retrotransposons preferentially accumulated in the *Abp* gene regions after lineage separation and roughly followed the pattern of *Abp* gene expansion. By contrast, the proportion of shared vs. lineage-specific ERVII repeats in the *Abp* region resembles the rest of the genome.

**Conclusions:**

We confirmed the role of L1 repeats in *Abp* gene duplication with the identification of recombinant *L1Md*_*T* elements at the edges of the most recent mouse *Abp* gene duplication. High densities of L1 and ERVII repeats were found in the *Abp* gene region with abrupt transitions at the region boundaries, suggesting that their higher densities are tightly associated with *Abp* gene duplication. We observed that the major accumulation of L1 elements occurred after the split of the mouse and rat lineages and that there is a striking overlap between the timing of L1 accumulation and expansion of the *Abp* gene family in the mouse genome. Establishing a link between the accumulation of L1 elements and the expansion of the *Abp* gene family and identification of an NAHR-related breakpoint in the most recent duplication are the main contributions of our study.

## Background

The origin of gene diversity has attracted great interest since Ohno’s proposal that gene duplications create new genetic material [1,2]. Subsequently, numerous gene families arising as a result of expansions from a single gene have been identified [3]. Sequencing of mammalian genomes, including primates, rodents and others shows that ~90% of genes have been preserved, usually as single copies without duplication, disruption or deletion, since their last common ancestors. The remaining ~10% of genes are subject to frequent duplication, deletion and pseudogene formation [4-6]. These represent the volatile portion of the mammalian gene complement and generally possess functions that differ from those of the conserved gene set. Gene families, such as those involved in chemosensation, reproduction, host defense and immunity, and toxin metabolism that are expanded, usually as tandem duplications, in one lineage are often expanded in another. This is very likely because they confer similar evolutionary responses to similar environmental challenges. As expected in a scenario in which selection pressures favor an increase in copy number, these expanded gene families often show the footprints of positive selection in elevated ratios of nonsynonymous to synonymous nucleotide substitutions (*dN/dS*; sometimes reported as the rate Ka/Ks; [7]) in their coding regions [8-12]. Moreover, gene deletion and pseudogene formation events are rare, except among genes that have also been subject to duplication [4-6].

There are, however, important exceptions to the idea that gene families that are expanded in one lineage are similarly expanded in other lineages. In a study of the problem of separating derived from ancestral features of mouse and human genomes, Ponting and Goodstadt [13] reviewed the sequence properties of those mammal genomes and presented the following conclusions: 1) large numbers of rodent- or primate-specific genes lie within these greatly expanded groups of genes; 2) loss of ancient single-copy genes appears to have been rare, as were gains of new functional genes. Instead, most changes to the gene repertoire have occurred in large multicopy families; 3) the numbers and the sequences of such ‘environmental genes’ change, as adaptive responses to infection and other environmental pressures, including conspecific competition; and 4) the larger number of rodent-specific gene duplicates found in the mouse genome is almost entirely due to genes with roles in chemosensation, such as olfactory and vomeronasal receptors and pheromone genes.

Although one may infer the ultimate causes of the expansions of gene families from the functions of the genes themselves, the mechanisms underlying these expansions often are elusive. More than 50% of mammalian genomes are composed of retrotransposons, a heterogeneous group of transposable elements that can be divided into several families according to their origins [4-6,14]. Different families have been active during rodent evolution and the timing of their activity varies between lineages. Seven families have been active during the evolution of mice and rats: L1 (LINE), B2 (SINE), ERVI, ERVII, ERVIII and MaLR (LTR) [4]. The B1 (SINE) activity is mouse lineage-specific whereas ID elements (a family of SINEs) predominate in the rat lineage [4]. Because the elements of each family are numerous and have highly homologous sequences, they may cause misalignment and serve as homology breakpoints for non-allelic homologous recombination (NAHR). As a result, a gene between the homologous repeat sequences could be duplicated, deleted or inverted.

Interestingly, certain families of retrotransposons were found to be enriched at the junctions of segmental duplications in the human, bovine, mouse, rat and grapevine genomes [15-22]. Alu (SINE) elements appear to be the predominant repeat sequences at junctions in primates where they contribute to the interspersed pattern of gene duplication characterizing the human genome [15,17]. In the cattle and rodent genomes where tandem duplication predominates, junctions are enriched for L1 and LTR elements [16-18,20-22]. Moreover, L1 elements were found to mediate globin duplication in the ancestor of simian primates [23]. However, only ~12% of all human duplications resulted from misalignment of two repeat elements [22], refuting the importance of repeat sequences in the production of duplications. Alu repeats are preferentially associated with actively duplicating clusters on human chromosome 22 [24], suggesting that repeats accelerate evolution by gene duplication and thereby cause expansion and/or contraction of gene families [25]. Alu repeats have also been found associated with gene clusters on human chromosome 7 [26] and Mirs, a type of SINE repeat, are associated with an OR gene cluster on human chromosome 17 [27]. In mice and other non-human organisms, L1 elements are enriched throughout the regions surrounding the olfactory and vomeronasal receptors (ORs, V1Rs and V2Rs; [28]). Those authors could not, however, confirm the hypothesis that high densities of these L1 elements contributed to the expansions themselves. Nevertheless, the exceptional L1 densities in regions of considerably expanded gene families along with the knowledge that repetitive sequences are associated with an elevated recombination rate [29-33] and ectopic recombination [34-39] suggest that further investigation of this relationship is warranted.

For our study we chose the *Androgen*-*binding protein* (*Abp*) gene family because it expanded recently enough that some paralogs have identical sequences [40] and there is copy number variation between strains [41]. This gene region expanded independently in the mouse and rat genomes and to a much greater extent in the mouse genome than in the rat genome [40]. ABPs mediate assortative mate selection, based on subspecies recognition that potentially limits gene exchange between subspecies where they meet ([42,43] reviewed in [44]) and there is evidence that *ABP* constitutes a system of incipient reinforcement along the European hybrid zone where house mouse subspecies make secondary contact [45].

In this study, we examined the role of repeat element sequences in the expansions of the mouse and rat *Abp* gene families. We searched the *Abp* region for evidence that retrotransposons contributed to the gene family expansions, possibly serving as the substrates for NAHR. We further characterised the pattern of accumulation of repeats in the *Abp* region, forging a putative link between accumulation of retrotransposons and *Abp* gene family expansion.

## Methods

### Dot plot analysis

In order to capture recent duplication events, a dot plot analysis was produced for the *Abp* gene family regions of the mouse and rat. The UCSC genome browser (http://genome.ucsc.edu/; [46]) was used to obtain the sequence of the *Abp* region for the mouse from the NCBIM37 (mm9) genome assembly and the sequence of the *Abp* region for the rat from the Baylor 3.4 (rn4) assembly. Coordinates of *Abp* genes for the two rodents (Additional files 1 &2 in [40]) were used to define the boundaries of the whole gene family region using the UCSC genome browser. The span of the whole region was selected in order to subsequently split the region into 50 kb non-overlapping bins (Table 1). We produced the dot plot matrix for the region in both genomes using the BLASTZ program [47] and plotted the results using R project code (http://www.R-project.org/; [48]). We used these dot plots to search for patterns of recent duplication events. Repeat content viewed on the dot plot was retrieved from the “rmsk” table downloaded from the UCSC genome browser. This table represents an output of the RepeatMasker program (http://www.repeatmasker.org/; [49]).

**Table 1:** **Location of the mouse and rat *Abp *gene families**^**1**^

**Species**	**Chromosome**	**Region beginning**	**Region end**	**Regions size (Mb)**	**Gaps in assembly (bp)**
*Mus*	7	32000001	35000000	3	0
*Rattus*	1	86300001	86650000	0.35	21065

^1^(NCBIM37) and rat genomes (Baylor 3.4). Start and end coordinates identified to enable splitting of the regions into 50 kb bins.

### Analysis of repeat density

We assessed the densities of eight repeat families active in rodent lineages: L1, B1, ID, B2, ERV1 (ERVI), ERVK (ERVII), ERVL (ERVIII), MaLR [4,6] in the *Abp* gene family region. Although, we are aware that the contribution by size may not implicitly correlate with the actual counts, we assumed that the total sequence contribution of each repeat family matters more than the actual counts of repeats in the region under consideration. Therefore, we characterized the density, defined as coverage by repeats of a particular repeat family in base pairs (bp). Density, rather than counts, was also assessed in many previous studies (e.g. [5,6]). All the information on the distribution of repeats and their positions in the mouse genome was retrieved from the rmsk table (see above).

We divided the *Abp* gene family region into 50 kilobase (kb) non-overlapping bins to capture the mean and median repeat densities and their variability within the *Abp* regions. For the density of repeats in the gene family region, we assumed that the densities before the expansion of the gene family corresponded to the densities in the flanking regions. Thus, as a reference, we compared the densities in the *Abp* gene family regions with 50 kb non-overlapping bins in one megabase (Mb) segments proximal and distal to them. We excluded regions containing assembly gaps according the UCSC genome browser from the analysis if they covered more than 20% of the bin. Otherwise, we normalized the repeat density within the 50 kb bin according to the following formula: (coverage in bp) × (50,000 / (50,000 – gaps in bp)). We used the Mann–Whitney U-test to contrast bins from within the *Abp* region against bins of downstream and upstream sequences on either side of these regions and we applied the Bonferroni correction to account for multiple comparisons. All analyses were performed in the statistical environment R.

### Relative dating of gene family expansions

We retrieved intronic sequences of *Abp* genes from mouse and rat genome sequences for use in dating the expansion of their *Abp* gene families (Additional file 3). We used intron b for all mouse and rat sequences. Sequences were aligned using the MUSCLE multiple alignment tool [50,51] and pairwise differences were calculated using the APE package for the R-project [52]. We used the Kimura 2-parameter model with Gamma correction as a substitution model.

### Species specificity of L1 and ERVII repeats

To assess whether the accumulation of repeats in the *Abp* gene family regions is species specific (i.e. the repeats accumulated after the split of the lineages leading to mouse and rat), we divided repeat subfamilies (as defined by RepeatMasker) in the mouse and rat genomes according to whether they are shared between these two genomes or are unique for each of them. The subfamily was considered as specific to one lineage if no copy of that subfamily was found in the other genome. Repeats that belong to species-specific subfamilies thus likely accumulated after the split of the mouse and rat lineages, whereas those belonging to shared subfamilies resided within the gene family regions before the split of the two species. The proportions of density contributed by lineage-specific and lineage-shared repeats in the *Abp* gene family regions were compared to the autosomal-wide proportion of these repeats.

### Repeat accumulation along evolutionary time

Repeat sequences in the *Abp* gene family region in both genomes were divided into bins by 1% of their divergence from consensus obtained from the rmsk table (to get a percentage divergence, the millidiv numbers in the table were divided by 10). Fold change in density of repeats contributed by specific and shared repeat subfamilies (see above) was subsequently plotted by divergence bin. The fold change represents how many times the density of repeats in a given divergence bin is higher or lower than the average autosomal-wide density (the same density equals 1) in non-overlapping windows of the same size as the size of the *Abp* region (e.g. for the *Abp* region in the mouse genome, the autosomal genome was split into 3 Mb windows). Windows containing assembly gaps involving more than 10% of the sequence were excluded from this analysis. The distributions were subsequently compared to pairwise half distances between intronic sequences of each gene family (see above).

## Results

### Identification of duplication events in the Abp region

**Figure 1:**
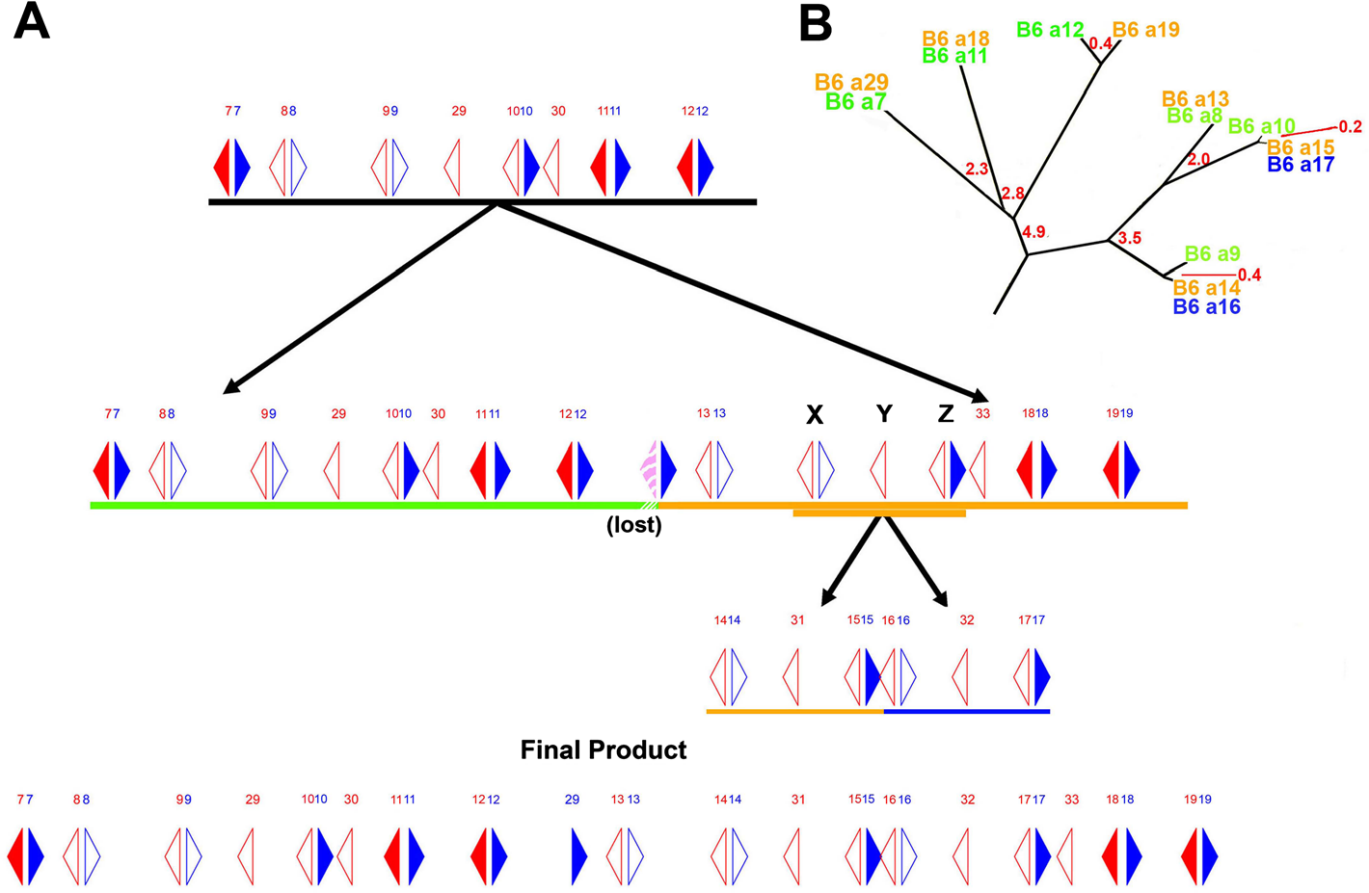
**A model for recent *Abp *gene duplication events.** (**A**) Partial map of *Abp* genes with arrows depicting genes (*Abpa* in blue, *Abpbg* in red); solid filled arrows are complete (potentially expressed) genes, while open arrows are putative pseudogenes (modified from [41]). <*Abpbg*-*Abpa* > modules are numbered *7*–*19* above the linkage map. Two duplications are depicted: 1) a large block of genes that duplicated to create the products underlined in green and orange; 2) those paralogs/modules designated X, Y and Z in the block on the right created products that are underlined in orange (*14*-*31*-*15*) and blue (*16*-*32*-*17*). The center portion of the *Abp* gene region created by these two duplications is shown as “Final Product” at the bottom of (**A**). (**B**) A phylogeny of the genes in the two duplications shown in **A** (modified from [40]). The partial *Abp* phylogeny (panel **B**) was modified from Laukaitis et al. [40], who produced an NJ phylogeny of intron 2 from rodent *Abpa* genes (their Figure 3, yellow clade at the top of the figure). The *Abp* branches derived from the mouse genome (labeled B6) were retained and the other branches removed. Paralog products are in a typeface color matching the bars that designate their places in the duplications and age estimates in Panel **A**. The age estimates of the duplications calculated by Laukaitis et al. [40] were also retained and are shown in red typeface.

**Figure 2:**
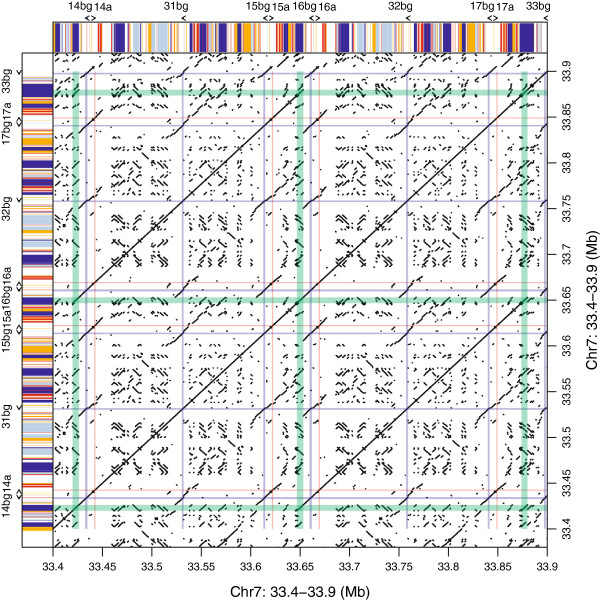
**Dot plot of the most recent duplication in the mouse *Abp *gene family region.** L1 (light blue, dark blue) and ERVII (red, orange) repeat content and orientation is diagrammed on the sides with different colours (L1 plus strand, light blue; L1 minus strand, dark blue; ERVII plus strand, red; ERVII minus strand, orange). For the recent *Abp* gene family duplication of *14*-*31*-*15* and *16*-*32*-*17*, genes are depicted with coloured lines (*Abpbg*, red; *Abpa*, blue). *L1Md_T* repeat elements on the edges of the duplicated blocks are marked with green lines.

**Figure 3:**
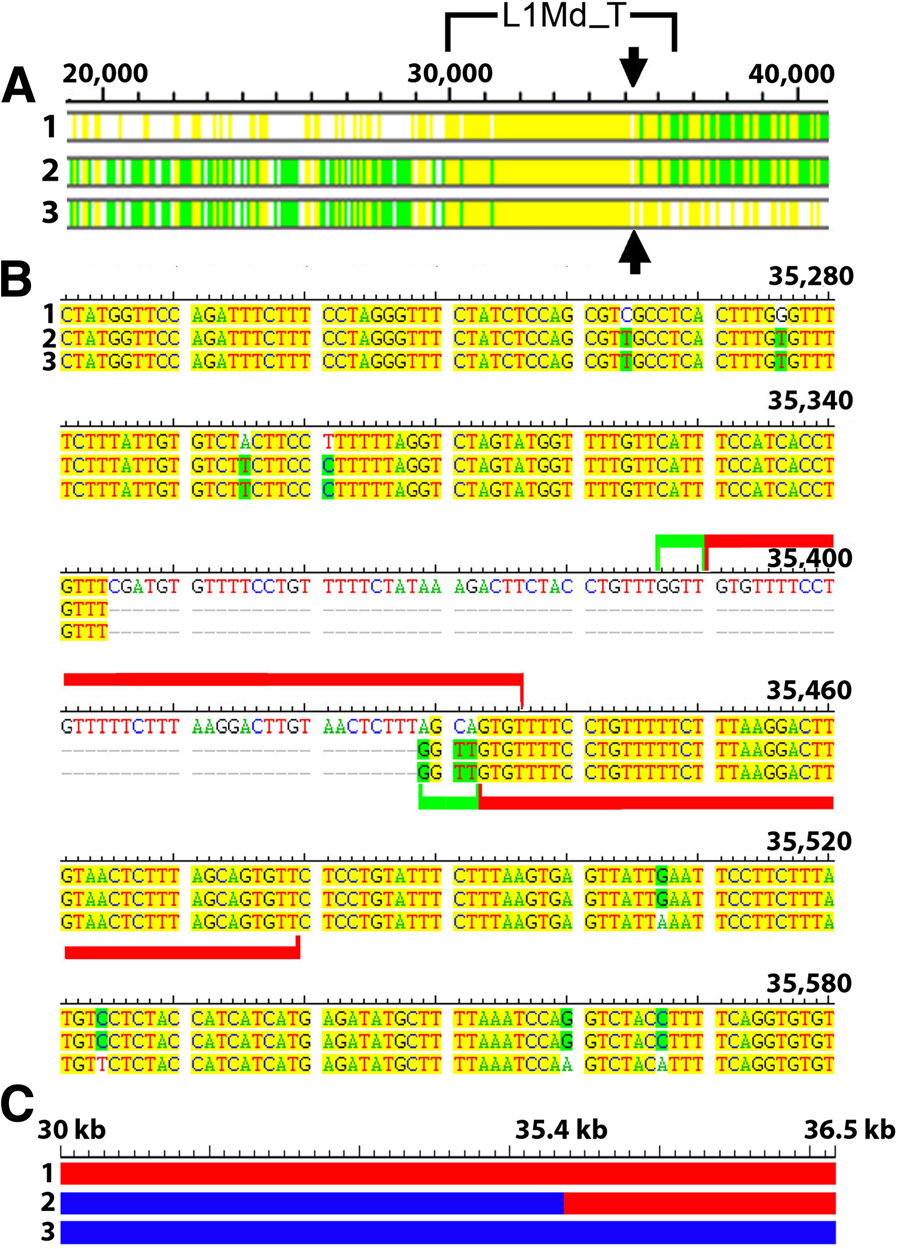
**Breakpoints for the most recent *Abp *gene duplication. A**) The region of alignment in all three sequences between ~30 kb and ~36 kb includes the nearly full-length *L1Md*_*T* sequence. The aligned sequences are shown as bars with the *L1Md*_*T* element set off by a bracket above the bars. Bar 1: the 33 kb region immediately to the left of *Abpbg14p*; bar 2: the *L1Md*_*T* repeat on the right flank of *Abpa15*; and bar 3: the region containing the *L1Md_T* repeat to the right of *Abpa17*. Bars in the upper part of the figure show the alignment of the three sequences over slightly more than 20 kb. Regions that align in all three sequences are tinted yellow; regions that align in two of three are tinted green and regions that do not align in any of the three are untinted. **B**) An alignment of 360 bp of the three sequences that surround the gap shown by black arrows in panel A. Two duplications that occur within the gap are depicted with red bars. We bracketed in green a GGTT preceding the rest of the duplication, which is marked with a red bar/bracket. We note that there are also shorter indels, e.g., TGTGTTTTCCTGTTTTTC, within the gap. Proximal to and at four nucleotides in the gap (GGTT), sequences 2 and 3 are identical (i.e. seven of eleven divergent sites shown in the figure). However, distal to the gap, 2 is identical to 1, while 3 differs at four divergent sites. **C**) Bars representing the entire *L1Md_T* sequences 1, 2 and 3 show that, for 384 divergent sites proximal to the breakpoint, 2 is identical to 3; for 127 divergent sites following the breakpoint 2 is identical to 1.

Because of the tandem structure of the duplications in the *Abp* gene regions, we used dot plots to identify duplication blocks and breakpoints (Additional files 1 and 2). Two large duplications occurred recently in the mouse *Abp* region, increasing the number of < *Abpbg*-*Abpa* > modules several times (Figure 1). The most recent event occurred <200,000 years ago [40]. In this event, one block of genes duplicated to produce the genes < *Abpbg*14p-*Abpa*14p > *Abpbg*31p < *Abpbg*15p-*Abpa*15 > (hereinafter abbreviated *14*-*31*-*15*) and < *Abpbg*16p-*Abpa*16p > *Abpbg*32p < *Abpbg*17p-*Abpa*17 > (abbreviated *16-32*-*17*). Figure 2 shows a dot plot of the *14*-*31*-*15* and *16*-*32*-*17* gene duplication blocks diagramed in Figure 1. The green lines flanking the diagonal *Abp* gene blocks contain the dots representing L1 elements belonging to a very young L1 subfamily (*L1Md*_*T*) that apparently misaligned to cause the duplication. The specific breakpoints are shown in Figure 3 and are described in detail below. Tandem duplications preceding the two large duplications were older and their structures were considerably eroded by deletions and insertions of younger elements.

### Analysis of the repeat sequences in the Abp block duplication

We identified the *L1Md*_*T* sequences on the left flank of *bg14p*, between *a15* and *bg16p*, and on the right flank of *a17*. These are shown within the sets of green lines in Figure 2. We aligned 20–30 kb surrounding these *L1Md*_*T* sequences and examined the alignment for putative breakpoints. The best candidate region is shown in Figure 3 where Panel A shows a complete alignment of the three *L1MD*_*T* sequences with a putative breakpoint identified. This candidate breakpoint is shown in detail in Figure 3B wherein a 50 bp sequence is repeated on the left flank of *bg14p*, but appears in only one copy in the *L1Md*_*T* sequences on the right of *a15* and *a17*. Thus, in the *a15* and *a17* sequences in this region, the missing repeated sequence plus 42 bp preceding it constitute an 84 bp “gap” in sequences 2 and 3 (Figure 3B). We suggest that this unequal distribution of the duplicated 50 bp sequence is consistent with the notion of a misalignment that created the breaks that resulted in the duplication of the ancestral segment to produce the *14*-*31*-*15* and *16*-*32*-*17* segments (Figure 1). This is supported by eleven divergent sites shown in Figure 3B. Sequences 2 and 3 are identical at four divergent sites proximal to, and three divergent sites within the gap just described. Beyond (distal to) the gap, sequences 1 and 2 are identical at four divergent sites. These patterns are consistent at 384 divergent sites upstream and 127 divergent sites downstream of the mis-paired AGCA/GGTT shown in the gap, and they support the idea that this is the breakpoint for the duplication (Figure 3C). Returning to Figure 3B, we propose that the misalignment that created the break probably occurred during replication at the point where synthesis of a new strand had proceeded just to the end of the GGTT (in the first green bracket). If a hairpin loop formed in strand 1 at that moment, the newly synthesized GGTT end might have slipped ahead to line up with the AGCA further downstream. This mismatch could have been stabilized by the TTT ahead of both tetra-nucleotides and the continuing synthesis of the duplicated 51 bp of sequence beyond them. In fact, slippage would be expected in the gap sequence because it is rich in a core unit of G followed by three or more Ts. In any event, destabilization at the mis-paired AGCA/GGTT (second green bracket) would then set the stage for the impending break by which NAHR produced the duplication of the *Abp* genes in this region. There may be alternative explanations but the important point is that the result was to switch strands at the AGCA/GGTT breakpoint as shown by the pattern of divergent sites we described above.

### The size of NAHR-duplicated Abp blocks varies considerably

A more detailed exploration of the entire *Abp* gene region clarified the proposal of Karn and Laukaitis [41] for the majority of genes in the center of the 3 Mb region. It is apparent from our dot plot analyses that the *14*-*31*-*15* and *16*-*32*-*17* duplication was preceded by a duplication of a much larger gene block containing the progenitors of what are now < *Abpbg*-*Abpa* > modules *7*, *8*, *9*, *10*, *11* and *12*, as well as the single *Abpbg* pseudogenes *29* and *31*. This large block duplicated to create all the genes identified in the region shown in Final Product (bottom of Figure 1A) except the < *Abpbg*X-*Abpa*X > *Abpbg*Y < *Abpbg*Z-*Abpa*Z > progenitor of *14*-*31*-*15* and *16*-*32*-*17*. Thus, this original product accounted for all the other genes from the < *Abpbg*7-*Abpa*7 > to the < *Abpbg*19-*Abpa*19 > modules. In this process, the *Abpbg*29 gene in the original < *Abpbg*29-*Abpa*29 > module in the center of the duplication product was eliminated. Figure 1B shows the portion of the center *Abp* clade with genes not described in this duplication removed. All the *Abpa* gene pairs arising from the duplication are directly related as predicted by this model.

### Repeat density in the Abp gene family regions

**Figure 4:**
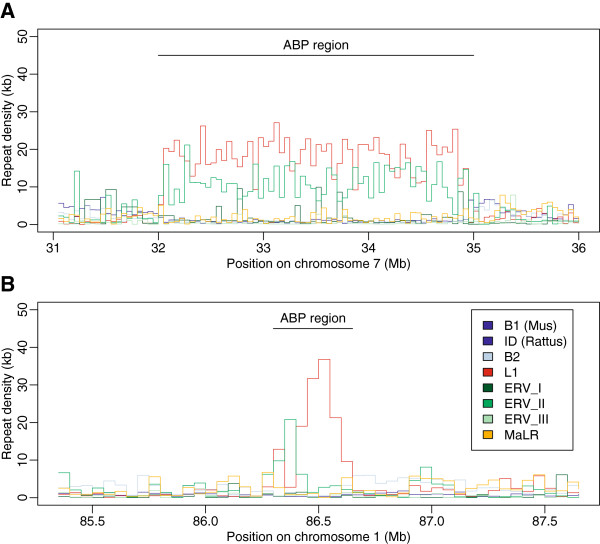
**Densities of repeat families on chromosome 7 in mouse (A) and chromosome 1 in rat (B) genomes within 50 kb windows plotted along the physical position of the chromosome in the *Abp *gene family regions and their one Mb flanking regions.** Densities are based on the RepeatMasker output provided at the UCSC Genome Browser website for mouse (NCBI M37/mm9) and rat (Baylor 3.4/rn4) genomes.

We contrasted the repeat density in 50 kb non-overlapping bins in the *Abp* gene family regions and in flanking regions one Mb proximal and distal to the gene regions (Figure 4; Additional file 3). ERVII and L1 repeat families were found to be in high densities in the *Abp* regions, whereas the other families (B1, B2, ID and MaLR) are depleted in these regions. The densities for the L1 repeats were more than ten times higher in the *Abp* regions than in the flanking regions and this family covered on average more than 30% of the 50 kb non-overlapping bins from the *Abp* region in both genomes. This pattern was statistically significant, for all elements in the mouse genome and for L1, ERVII and ID in the rat genome (Additional file 3).

### Age of L1 and ERVII repeats in the Abp regions

The L1 and ERVII subfamilies were divided according to whether they were shared between mouse and rat, or whether they were specific to one of the lineages. We compared the proportions of repeat density in the *Abp* region contributed by either the shared or lineage-specific groups (Table 2; Additional file 4). A considerable portion of the L1 repeat density in the *Abp* region (>90% in mouse, >80% in rat) is composed of young lineage-specific repeats, which contrasts with the genome-wide pattern for the L1 family where only about 50% of repeats are lineage-specific. The difference is statistically significant in both genomes (Table 2). The density of ERVII family repeats is almost equally distributed between lineage-specific and lineage-shared elements. Despite the significant difference between the ratios in the *Apb* region and flanking regions, ERVII density does not differ substantially from the genome-wide pattern (51% genome-wide vs. 57% in the *Abp* region; Table 2).

**Table 2:** **Proportions of lineage**-**unique and lineage**-**shared repeats in the *Abp *gene family regions**

**Species**	**Repeat family**	**Coverage (in bp) of**		**Proportion of unique repeats in the *Abp *region (CI)**	**Proportion of unique repeats genome-wide**	**P-value**^**1**^	**Significance**
		**Unique**	**Shared**				
mouse	L1	1004910	71236	0.93 (0.92-0.95)	0.52	0	***
	ERVII	378977	280161	0.57 (0.53-0.61)	0.51	0.002	**
rat	L1	96003	20038	0.83 (0.71-0.91)	0.52	0	***
	ERVII	15034	22521	0.40 (0.24-0.55)	0.42	1	n.s.

^1^Data resampling with Bonferroni correction (n = 4) used to obtain p-values.

**Figure 5:**
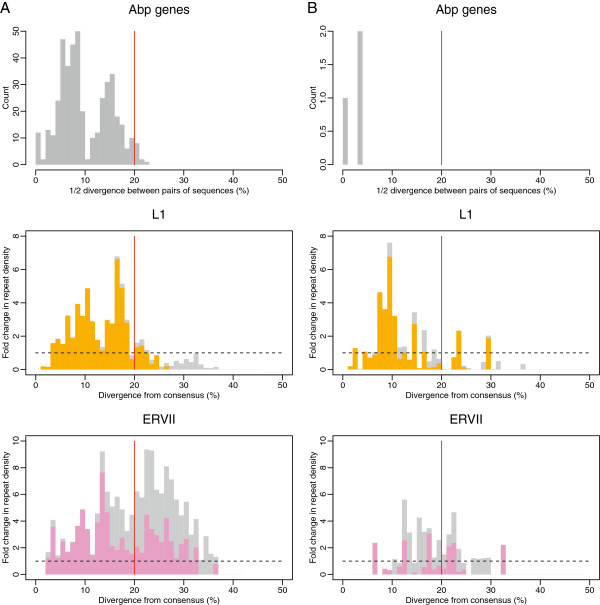
**Change in repeat density over time compared with the timing of *Abp *gene duplications.** The mouse genome is shown in (**A**) and the rat is in (**B**). Top panels show the approximate timing of *Abp* gene duplications. Fold-change in density of L1 (center) and ERVII (bottom) repeats are colored depending on whether they are contributed by unique (orange/violet) or shared (grey for both) repeat subfamilies. All are plotted along 1% divergence bins. Divergence for the two repeat families represents divergence from genome-wide consensus (i.e. approximate time when the element was inserted in the *Abp* region). Dating of the *Abp* gene duplications was based on the distribution of pairwise half distances between *Abp* genes using the Kimura 2-parameter mutation model. Given the pattern of unique and shared repeat subfamilies on the genome-wide scale for all repeat families, the mouse-rat split occurred around 20% of divergence from consensus (red line).

The accumulation of L1 and ERVII repeats in the *Abp* region was also analyzed in the context of their age (i.e. the approximate time that the repeats were inserted in the *Abp* region). We compared the distributions of fold-change in repeat density of lineage-specific and lineage-shared repeats given the divergence from consensus (Figure 5A, B – middle, bottom) with the actual distributions of pairwise distances for the *Abp* genes (Figure 5A, B – top). The fold-change in density of L1 and ERVII repeats unique for mouse and rat was the focus of our evaluation. We found that the rapid accumulation of L1 elements occurred after the split of the two lineages. The increased rate of accumulation (i.e. insertion) of L1 repeats in the mouse genome is characterized by two peaks that correspond to the distribution of the pairwise distances between *Abp* genes (Figure 5A, B – top, middle). The increased rate of accumulation in the rat genome was also lineage-specific, however, the correspondence between the L1 accumulation and the gene family expansion was not so clear. The pattern of ERVII retrotransposon accumulation differs from the L1 pattern in that it exhibits, on average, an increased rate of accumulation along almost its entire evolutionary history, with considerable accumulation even before the split of the lineages (Figure 5A, B – top, bottom).

## Discussion

The evolution of gene families is still poorly understood, despite the appearance of an ever-increasing number of sequenced genomes. Many gene families have expanded much faster than expected based on random gene gain and loss, and transposable elements (i.e. retrotransposons) and selection have been cited as main causes of gene family expansion and contraction [25]. Retrotransposons are associated with local recombination [29-33], perpetuate ectopic recombination [34-39] and are enriched at the breakpoints of segmental duplications in various organisms [16,17,21,22]. Because repeat elements also represent highly homologous sequences, high local densities may have caused instability and consequently an increase in the rate of NAHR, as proposed for Alu elements in the human genome [17]. While repeat enrichment at the junctions of segmental duplications is associated with only ~12% of all duplications [22], this subgroup may represent the tandem duplications responsible for active expansion of gene families, such as the Alu repeats on human chromosome 22 [24].

We focused on *Abp* genes because their expansions in the mouse and rat genomes occurred after the divergence of the two species. We searched the *Abp* regions for evidence that retrotransposons contributed to their gene expansions, perhaps acting as substrates for NAHR. Further, we investigated the patterns of retrotransposon accumulation in the *Abp* region and establish a link between their accumulation and the *Abp* gene family expansion.

### Identification of breakpoints and the evidence for retrotransposons in NAHR

Dot plot analyses suggest that retrotransposons served as substrates for NAHR in the most recent mouse *Abp* gene family region duplication. We identified *L1Md_T* elements at the edges of the most recently duplicated gene block of the *Abp* gene region, proposed to have occurred by NAHR [41]. Recombination after misalignment of the *L1Md*_*T* sequences flanking the 14-31-15 and 16-32-17 segments could have caused this duplication. Analysis of the three *L1Md*_*T* repeats implicated in this event reveals evidence of recombination between the two outer elements to create a hybrid middle repeat (Figure 3).

We sought additional breakpoints at the edges of older duplication blocks. Unfortunately these erode quickly as elements are being inserted and deleted. We suppose that the duplication we identified must have been very recent and it is even possible that they are not yet fixed in the population, consistent with findings of copy number variation in this region [41].

Karn and Laukaitis [41] suggested that there were two mechanisms involved in the duplications leading to the 64 *Abp* paralogs in the mouse genome: a slower one that produced paralogs in an inverse adjacent order as predicted by Katju and Lynch [53], and a faster one that proceeded by NAHR and produced paralogs in direct, not inverse, order. In this report, we identify *L1Md*_*T* elements on the flanks of the most recent *Abp* duplication block with breakpoints produced by NAHR. Thereby, we provide the details of the second mechanism and an example of NAHR causing duplication resulting in daughter gene blocks in direct, not inverse order.

### Density of the L1 and ERVII repeats in the Abp region

Identification of *L1Md*_*T* elements at the edges of the most recent *Abp* gene duplication in the mouse genome motivated us to further explore retrotransposons in the *Abp* gene family regions of mouse and rat. L1 and ERVII repeats are denser in the *Abp* regions than in the regions flanking them, with L1 repeat densities 10 times higher in the rodent *Abp* regions than in one Mb flanking regions. There are also sharp transitions between the high density of L1 and ERVII repeats within the *Abp* gene family region and the less dense flanking regions. The abruptness of these transitions shows that the high densities of L1 and ERVII are specific to the *Abp* regions and are not a general property of the genomic regions in which the *Abp* families reside. It is interesting that our observations of high L1 density in the *Abp* regions are similar to reports of high densities of L1 retrotransposons in the regions harboring V1R, V2R and OR receptor gene families of rodents and several other non-human organisms [28]. Similarly, others have found enrichment of retrotransposons (Alu and Mir elements) around human gene clusters [26,27]. All this supports the view that the association between higher retrotransposon densities and the duplicated nature of the *Abp* region is not coincidental.

Since L1 and LTR (including ERVII) repeat families are enriched at junctions of segmental duplications in the mouse and rat genome [16,18,21,22], one may speculate on the role of the increased density of repeat elements in gene family expansion. We want to know what drove the accumulation of retroelement repeats in the *Abp* gene regions. One possibility is that selection for increased gene copy number resulting from densely packed repeat elements is a cause of the association. However, there are alternative explanations wherein the higher densities of these repeats might have resulted indirectly from the duplicated nature of the *Abp* region. Among these are: 1) the repeats accumulated in the region passively along with newly-duplicated *Abp* genes; 2) as redundancy of gene function increased with continued *Abp* gene duplication, the region more readily accepted insertion of additional repeats; and 3) accumulation of repeat elements occurred because they contributed to the allelic regulation of particular genes [28]. In fact it is likely that multiple mechanisms led to the observed pattern of repeats.

### Timing of repeat accumulation

The approximate ages of retrotransposon accumulations were assessed by comparing ratios of the densities of lineage-specific vs. lineage-shared subfamilies in the *Abp* region with the genome-wide ratios (Table 2; Additional file 4). We found striking differences between L1 and ERVII repeat families in both the mouse and rat genomes. ERVII subfamilies were distributed approximately equally between lineage-specific and lineage-shared subfamilies in both genomes. This was not true for the L1 family where the majority of repeats (>90% in the mouse and >80% in the rat genome) were lineage-specific. Thus, >50% of ERVII repeat content originated from insertions that occurred near the ancestor of the *Abp* gene family, while almost no L1s were present in the *Abp* region before its expansion. Because we were concerned about the effect of gene conversion on our ability to time L1 accumulation, we also analyzed L1 repeats for gene conversion events which could obscure that timing and found evidence only of intra-, not inter-subfamily gene conversion events (data not shown). Intra-family gene conversion events may have occurred as part of the misalignment and recombination characterizing NAHR. In any event, these would not influence our ability to differentiate between unique and shared L1 repeats or to discern the overall pattern of repeat accumulation.

We dated the change in L1 and ERVII retrotransposon densities along evolutionary time and compared it with the time frame of *Abp* gene expansion inferred from pairwise distances (Figure 5). The change in L1 density over evolutionary time in the mouse genome occurs in two clear peaks composed of exclusively mouse lineage-specific repeats, which correspond to the distribution of pairwise distances of *Abp* genes. The correspondence between the pattern of gene duplication events and the date of repeat insertions in the mouse genome suggests that the two processes are related. This finding may be explained by three scenarios: 1) repeats are intrinsically involved in the gene family expansion process, 2) repeats accumulated immediately after the duplication to promote allelic regulation and 3) repeats accumulated as a result of an increase in tolerance due to an increase in redundancy. However, the third scenario is not likely because it predicts that the accumulation of repeats would not closely follow gene pairwise distances and would continue even after the burst of duplication. The first and second explanations cannot be sufficiently resolved with our data. However, the idea that *Abp* gene duplication results from accumulation of L1 elements seems most likely in light of the findings we report here.

The situation is quite different for the ERVII family. The rate of ERVII accumulation (fold change) in the *Abp* gene region along almost its entire evolutionary history is several times higher than expected from the genome-wide distribution, with considerable repeat insertion before the mouse and rat split. The high density of mouse and rat shared ERVII repeats (i.e. repeats inserted long before the *Abp* gene family expansion) leaves little doubt that their high densities resulted from passive duplication and/or an unconditionally higher tolerance of accumulation by the *Abp* gene region. The fact that these repeats were present at high density long before the *Abp* gene family expansion occurred makes the link between their higher densities and the expansion less clear.

## Conclusion

We have presented direct evidence for the contribution of a repeat element, *L1Md*_*T*, to the *Abp* gene region expansion. Our evidence includes identification of the breakpoint of a very recent duplication within a hybrid *L1Md*_*T* element, which strongly supports the supposition that this duplication occurred by NAHR [41]. We have explored the literature for an observation similar to the breakpoints in the *L1Md_T* elements that we describe here for the most recent duplication of *Abp* genes. The reports we found involved breakpoints producing duplications of genes or parts of genes in cancer and other diseases in somatic cells. These instances of NAHR are examples of mitotic recombinations producing clinically significant aberrations but not stable increases in gene copy number in the genome. To the best of our knowledge, the breakpoints we describe in repeat elements that have produced duplicated paralogs by meiotic NAHR in the germ line constitute a unique observation in studies of gene duplication.

We also found higher densities of L1 and ERVII retrotransposons along with depletion of other retrotransposon types occurring with abrupt transitions at the gene region boundaries, suggesting that the occurrence of these repeats is tightly associated with *Abp* gene duplication. We observed that the major contribution to the total L1 density occurred after the split of the two lineages in both genomes, with clear overlap between the accumulation pattern of L1 elements and the *Abp* gene family expansion, at least in the mouse genome. Regardless of whether the higher densities of L1 repeats are a cause or a consequence of the gene family expansion, this demonstrates the putative link between the accumulation of these elements and the gene family expansion. By contrast, the accumulation pattern of ERVII repeats is complex with a considerable portion of the total ERVII density predating the mouse-rat lineage split, similar to genome-wide patterns.

## Availability of supporting data

Data sets supporting the results of this article are included within the article and its Additional files.

## Competing interests

The authors declare that they have no competing interests.

## Authors’ contributions

VJ, CML and RCK conceived of the project, analyzed the data and wrote the manuscript. VJ performed bioinformatic analyses. RCK and CML performed detailed sequence analysis. All authors read and approved the final manuscript.

## Supplementary Material

Additional file 1 **Dot plots for the *Abp *gene family region in the mouse genome using coordinates from theNCBIM37/mm9 assembly.** Click here for file

Additional file 2 **A dot plot for the *Abp *gene family region in the rat genome using coordinates from the Baylor 3.4/rn4 assembly.** Click here for file

Additional file 3 Average and median repeat densities in base pairs across 50 kb windows in the gene family (GFR) and flanking regions (FR). Click here for file

Additional file 4 **Proportions of densities of elements belonging to lineage-shared (dark grey) and lineage-specific (light-grey) L1 and ERVII subfamilies in the *Abp *gene family region compared to the autosomal-wide ratio for these two families.** The subfamilies were divided according to whether they were shared between mouse and rat genomes or whether their presence was specific for one of them. Click here for file
