# Supplementary figures and images for: The role of retrotransposons in gene family expansions: insights from the mouse Abp gene family
Source: BMC Evol Biol. 2013 May 29;13:107. doi: 10.1186/1471-2148-13-107 (PMC3669608; doi:10.1186/1471-2148-13-107)

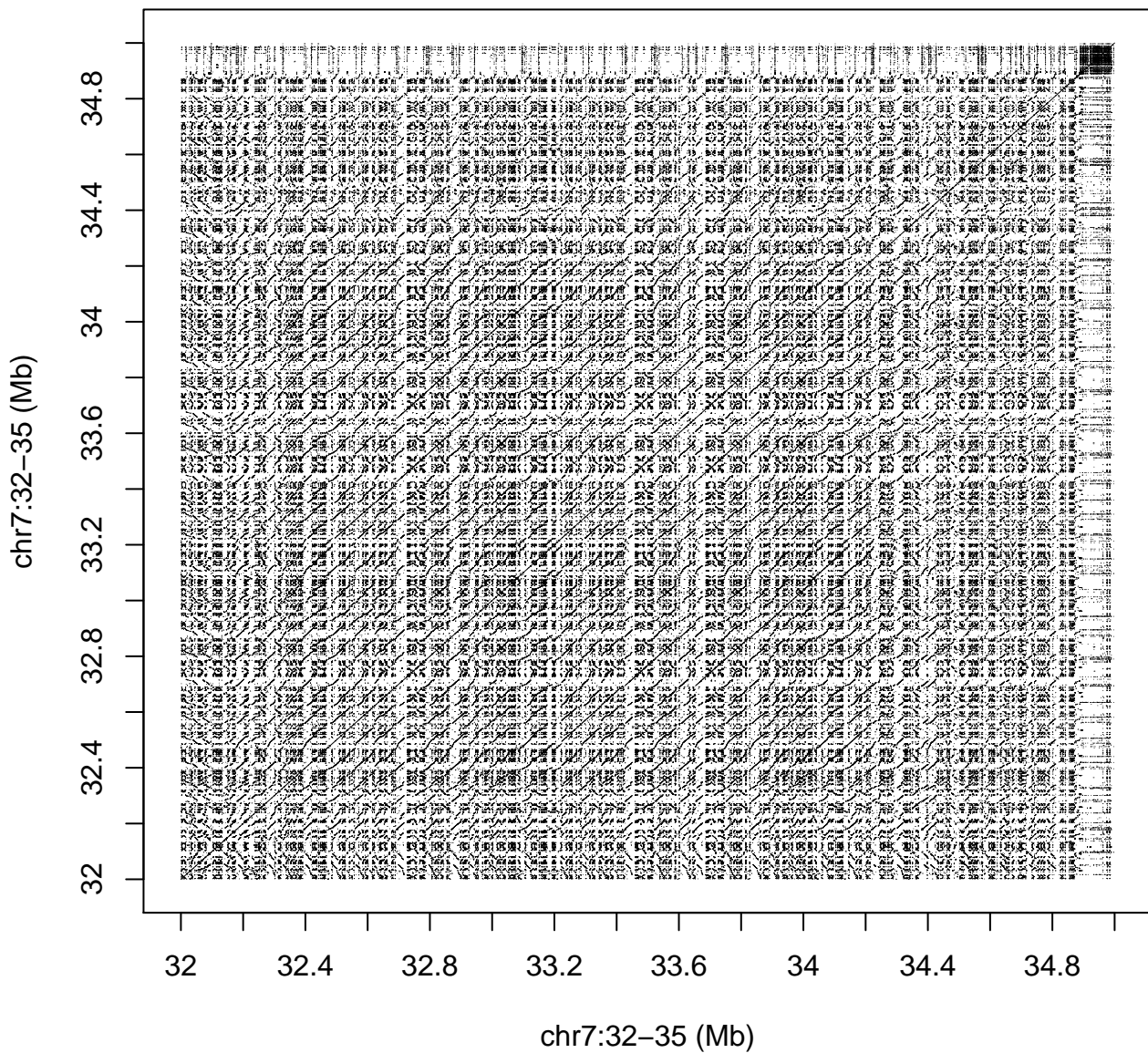

Supplement: Additional file 1 — Dot plots for the Abp gene family region in the mouse genome using coordinates from theNCBIM37/mm9 assembly. [file 1471-2148-13-107-S1.pdf]

chr4:59.9–61.9 (Mb)

60.1

59.9

59.9

60.1

chr4:59.9–61.9 (Mb)

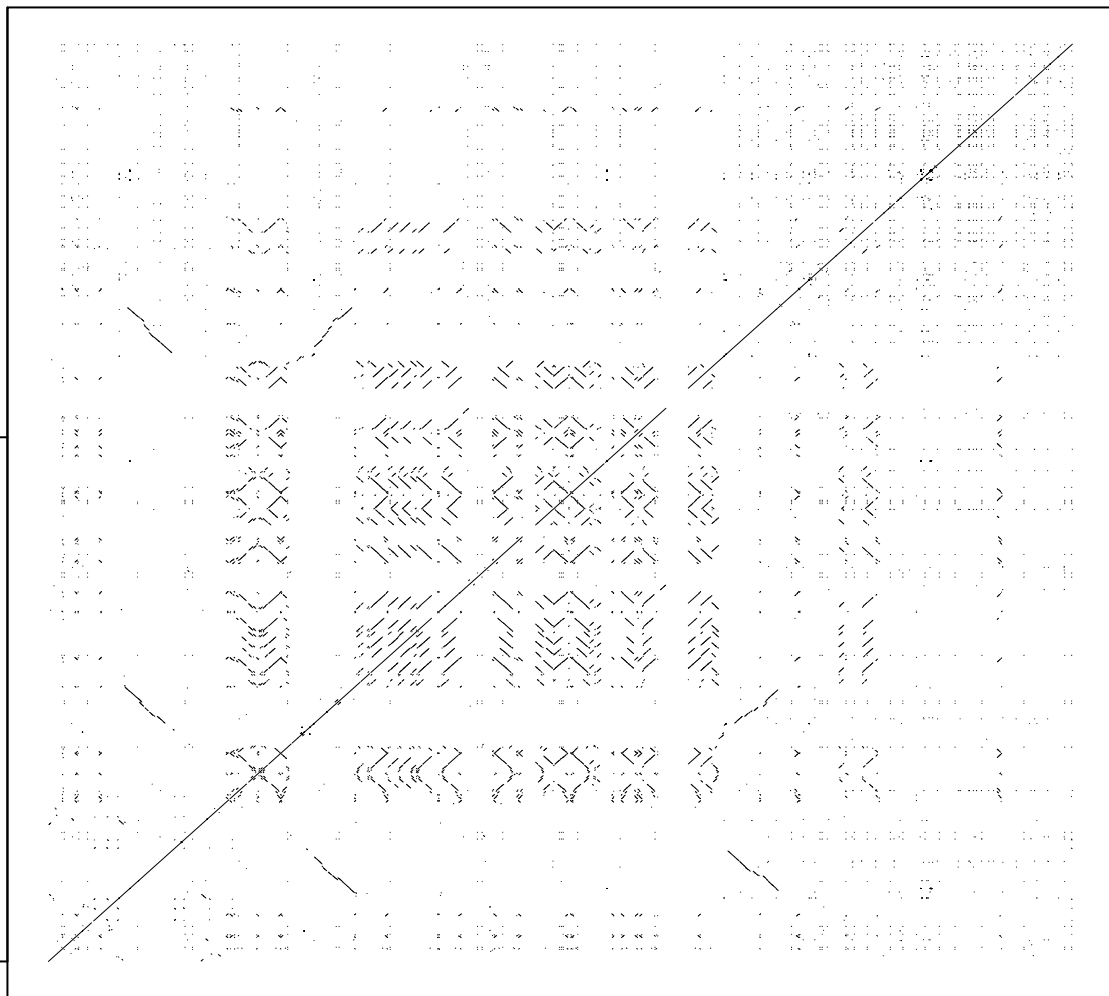

Supplement: Additional file 2 — A dot plot for the Abp gene family region in the rat genome using coordinates from the Baylor 3.4/rn4 assembly. [file 1471-2148-13-107-S2.pdf]

Mouse:L1

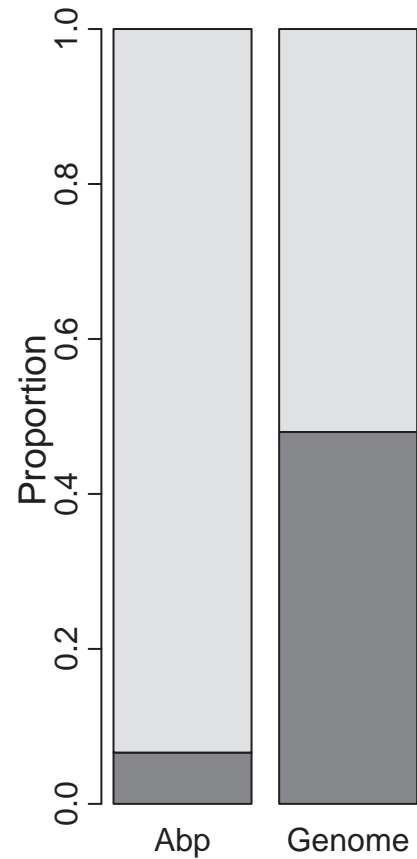

Mouse:ERVII

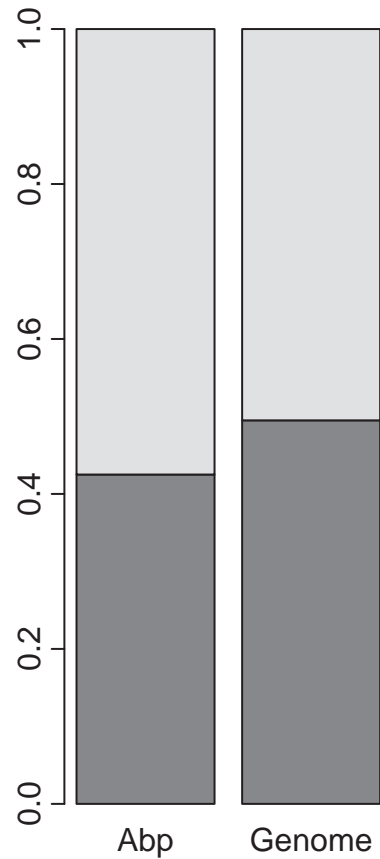

Rat:L1

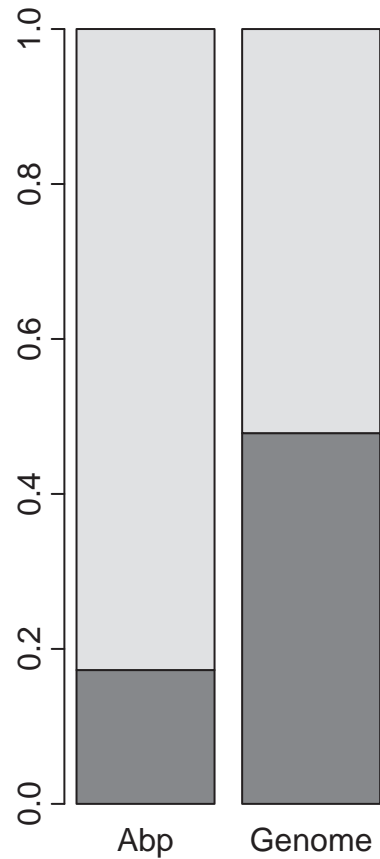

Rat:ERVII

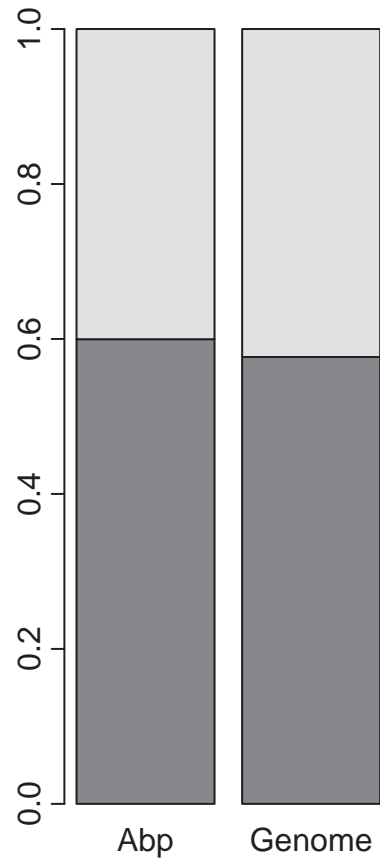

Supplement: Additional file 4 — Proportions of densities of elements belonging to lineage-shared (dark grey) and lineage-specific (light-grey) L1 and ERVII subfamilies in the Abp gene family region compared to the autosomal-wide ratio for these two families. The subfamilies were divided according to whether they were shared between mouse and rat genomes or whether their presence was specific for one of them. [file 1471-2148-13-107-S4.pdf]
