# Supplementary material for: The role of retrotransposons in gene family expansions: insights from the mouse Abp gene family
Source: BMC Evol Biol. 2013 May 29;13:107. doi: 10.1186/1471-2148-13-107 (PMC3669608; doi:10.1186/1471-2148-13-107)
Supplement: Additional file 3 — Average and median repeat densities in base pairs across 50 kb windows in the gene family (GFR) and flanking regions (FR). [file 1471-2148-13-107-S3.doc]

| Additional file 3. Average and median repeat densities in base pairs across 50 kb windows in the gene family (GFR) and flanking regions (FR) | | | | | | | | | | |  |
| --- | --- | --- | --- | --- | --- | --- | --- | --- | --- | --- | --- |
| 1. Mouse (NCBIM37/mm9) genome | | | | | | | | | | |  |
| Repeat Family | Flanking Regions (FR) | | Gene Family Regions (GFR) | | Ratio GFR/FR | | | | P-value^1^ | Significance^2^ | |
|  | Mean (%) | Median (%) | Mean (%) | Median (%) | Mean | | Median | |  |  |  |
| B1 | 3465.43 (6.93) | 3252.5 (6.51) | 900.68 (1.8) | 754.5 (1.51) | 0.26 | | 0.23 | | 1.67E-014 | *** | |
| B2 | 2660.25 (5.32) | 2604.5 (5.21) | 810.78 (1.62) | 669 (1.34) | 0.30 | | 0.26 | | 4.34E-013 | *** | |
| ERVI | 785.85 (1.57) | 0 (0) | 1376.47 (2.75) | 707 (1.41) | 1.75 | | N/A | | 2.63E-003 | ** | |
| ERVII | 2023.98 (4.05) | 914 (1.83) | 10985.65 (21.97) | 10859.5 (21.72) | 5.43 | | 11.88 | | 1.36E-013 | *** | |
| ERVIII | 691.33 (1.38) | 293.5 (0.59) | 287.88 (0.58) | 114 (0.23) | 0.42 | | 0.39 | | 1.08E-003 | ** | |
| L1 | 1369.48 (2.74) | 988.5 (1.98) | 17935.77 (35.87) | 18999 (38) | 13.10 | | 19.22 | | 1.25E-015 | *** | |
| MaLR | 2859.05 (5.72) | 2719 (5.44) | 1480.88 (2.96) | 1185 (2.37) | 0.52 | | 0.44 | | 4.71E-004 | *** | |
| Total | 13855.35 (27.71) | 10772 (21.54) | 33778.12 (67.56) | 33288 (66.58) | - | | - | | - | - | |
| 1. Rat (Baylor 3.4/rn4) genome | | | | | | | | | | |  |
| Repeat Family | FR | | GFR | | Ratio GFR/FR | | | P-value^1^ | | Significance^2^ | |
|  | Mean (%) | Median (%) | Mean (%) | Median (%) | Mean | Median | |  |  |  |  |
| B2 | 2773.26 (5.55) | 2573 (5.15) | 1886.57 (3.77) | 971 (1.94) | 0.68 | 0.38 | | 7.99E-001 | | n.s. | |
| ERVI | 456.4 (0.91) | 0 (0) | 493.71 (0.99) | 111 (0.22) | 1.08 | N/A | | 1.00E+000 | | n.s. | |
| ERVII | 1964.09 (3.93) | 1099 (2.2) | 5318 (10.64) | 2510 (5.02) | 2.71 | 2.28 | | 3.50E-001 | | ** | |
| ERVIII | 516.4 (1.03) | 408 (0.82) | 440.57 (0.88) | 474 (0.95) | 0.85 | 1.16 | | 1.00E+000 | | n.s. | |
| ID | 820.49 (1.64) | 811 (1.62) | 428.14 (0.86) | 292 (0.58) | 0.52 | 0.36 | | 2.35E-002 | | * | |
| L1 | 1613.51 (3.23) | 1012 (2.02) | 16577.29 (33.15) | 18024 (36.05) | 10.27 | 17.81 | | 2.42E-003 | | ** | |
| MaLR | 2640.2 (5.28) | 2370 (4.74) | 1459.29 (2.92) | 940 (1.88) | 0.55 | 0.40 | | 5.97E-001 | | n.s. | |
| Total | 10784.34 (21.57) | 8273 (16.55) | 26603.57 (53.21) | 23322 (46.64) | - | - | | - | | - | |
| ^1^Mann-Whitney U-test with Bonferroni correction (n=7)  ^2^*p=0.05-0.01; ** p=0.01-0.001; *** p<0.001 | | | | | | | | | | |  |
